# Supplementary material for: Power Outage: A Simulation Case for Anesthesiology Residents
Source: MedEdPORTAL. 2025 May 6;21:11523. doi: 10.15766/mep_2374-8265.11523 (PMC12052912; doi:10.15766/mep_2374-8265.11523)
Supplement: Supplementary file 1 — Simulation Case.docxSimulation Case Equipment.docxDebriefing Materials.pptxPostsimulation Survey.docx [file mep_2374-8265.11523-s001.zip › B. Simulation Case Equipment.docx]

**Appendix B: Simulation Case Equipment**

The following is a list of equipment and disposables that we found necessary to run this simulation successfully:

1. Manikin: Laerdal SimMan3G
2. Anesthesia machine without volatile agents except oxygen and medical air
3. Ambu bag/mask on the back of the anesthesia machine
4. Blood pressure cuff, SpO2 finger probe, ECG leads hooked up to the anesthesia machine
5. Mock anesthesia cart with anesthesia disposables (ETT, laryngoscope blades, syringes, etc) and an array of mock anesthesia medications including propofol and norepinephrine infusions.
6. Infusion pumps.
7. Battery-powered, portable patient monitor
8. OR drapes, clamps, chlorhexidine scrubs
9. PPE: assorted sterile gloves, gown, hats
10. Videolaryngoscopy with a Glidescope and a flexible fiberoptic bronchoscope. If a simulation lab doesn't have access to this technology, direct laryngoscopy may be employed. Video and fiberoptic airway equipment is not required for this scenario.
11. Adult code cart with defibrillator and travel oxygen tank
