# Supplementary material for: Power Outage: A Simulation Case for Anesthesiology Residents
Source: MedEdPORTAL. 2025 May 6;21:11523. doi: 10.15766/mep_2374-8265.11523 (PMC12052912; doi:10.15766/mep_2374-8265.11523)
Supplement: Supplementary file 1 — Simulation Case.docxSimulation Case Equipment.docxDebriefing Materials.pptxPostsimulation Survey.docx [file mep_2374-8265.11523-s001.zip › C. Debriefing Materials.pptx]

## Slide 1
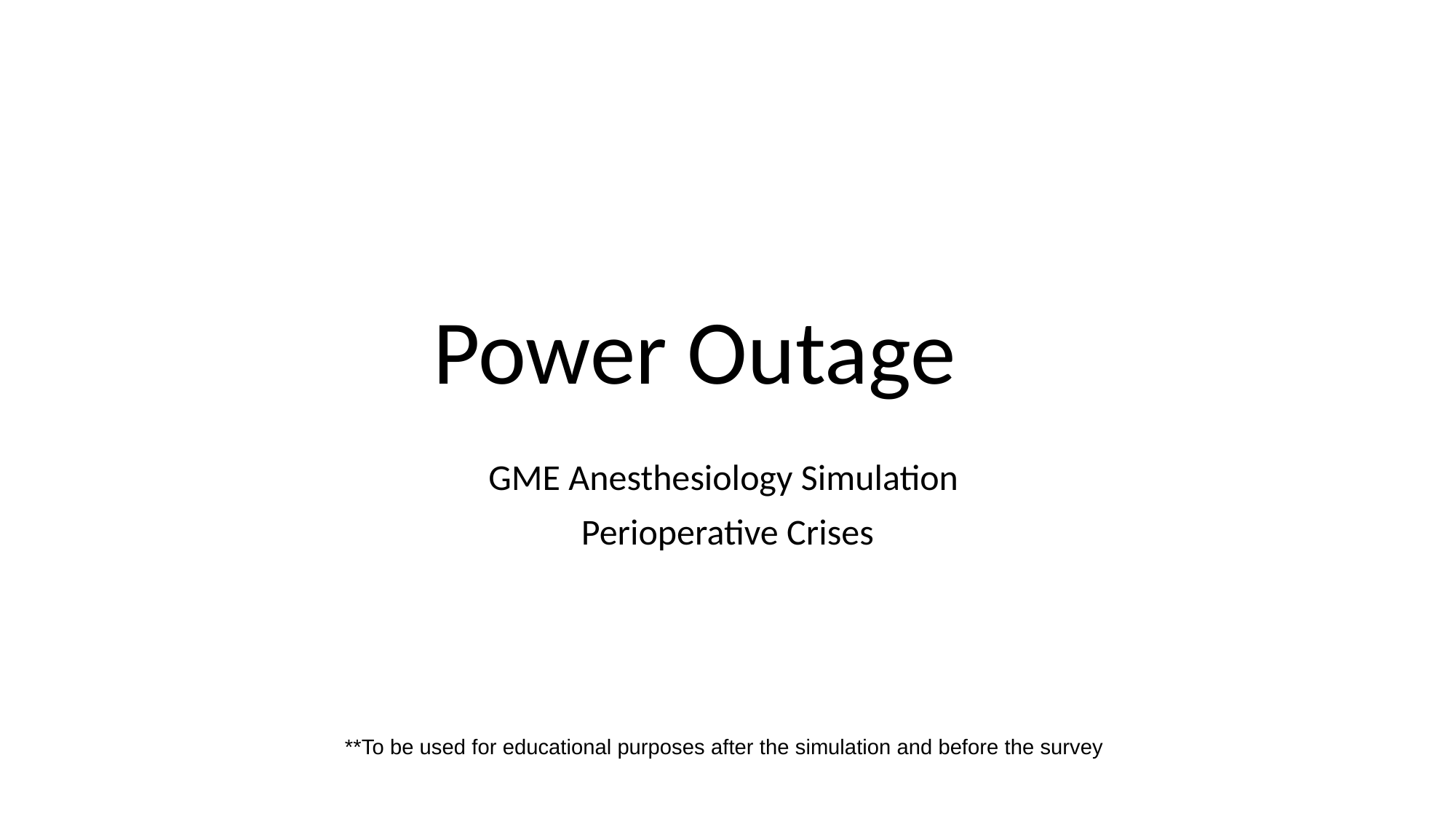

# Power Outage
GME Anesthesiology Simulation
Perioperative Crises
**To be used for educational purposes after the simulation and before the survey

## Slide 2
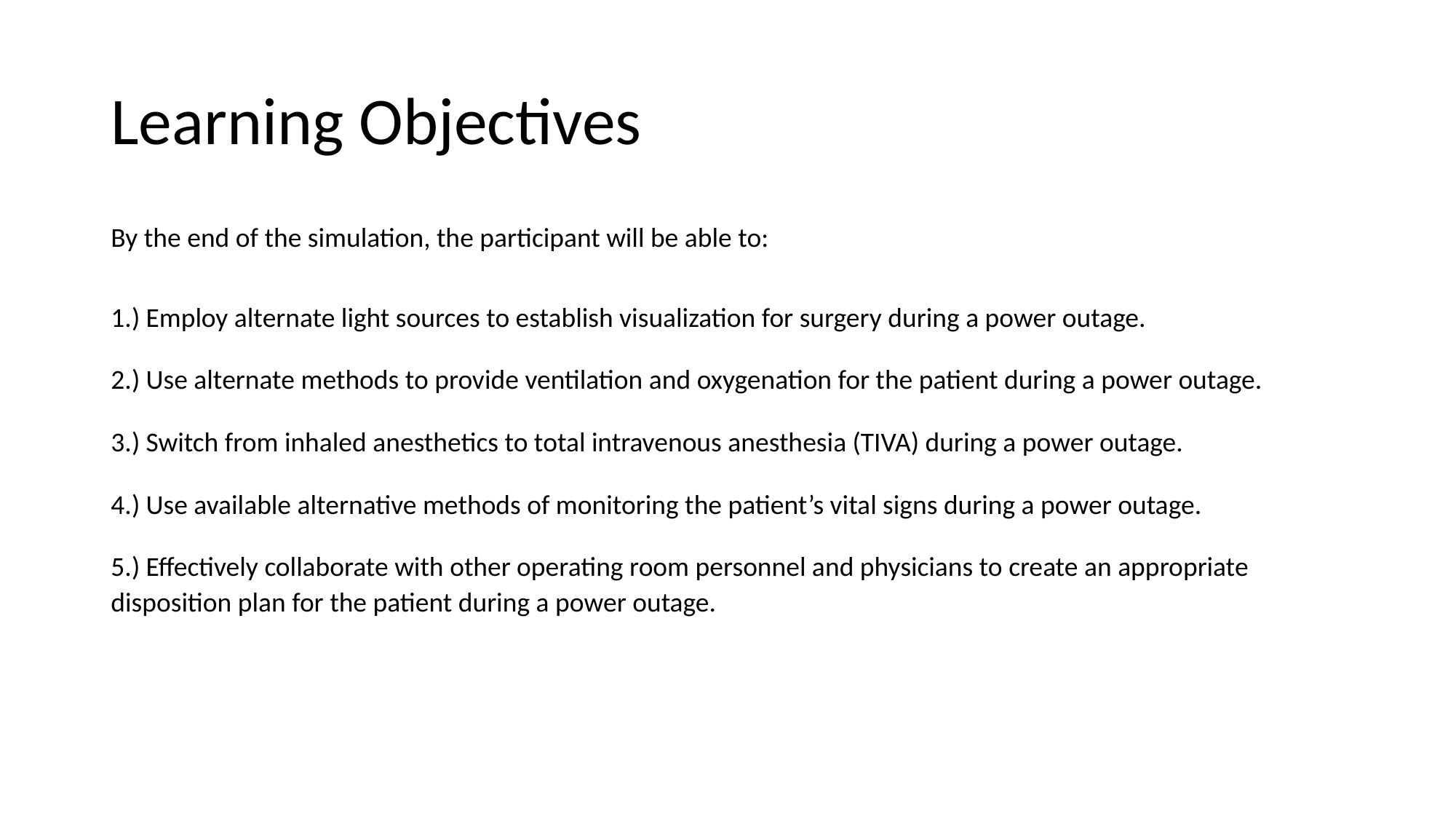

# Learning Objectives
By the end of the simulation, the participant will be able to:
1.) Employ alternate light sources to establish visualization for surgery during a power outage.
2.) Use alternate methods to provide ventilation and oxygenation for the patient during a power outage.
3.) Switch from inhaled anesthetics to total intravenous anesthesia (TIVA) during a power outage.
4.) Use available alternative methods of monitoring the patient’s vital signs during a power outage.
5.) Effectively collaborate with other operating room personnel and physicians to create an appropriate disposition plan for the patient during a power outage.

## Slide 3
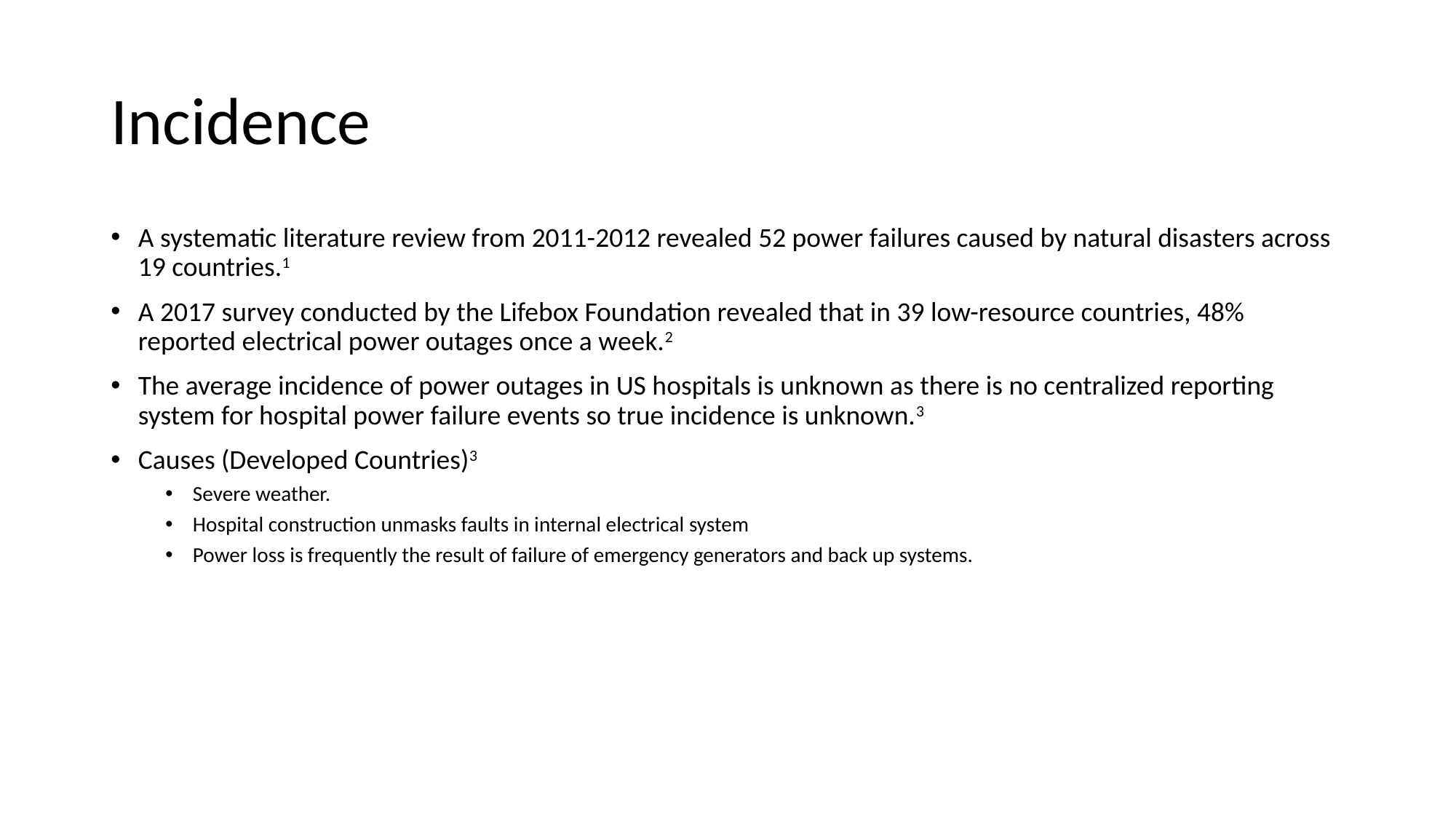

# Incidence
A systematic literature review from 2011-2012 revealed 52 power failures caused by natural disasters across 19 countries.1
A 2017 survey conducted by the Lifebox Foundation revealed that in 39 low-resource countries, 48% reported electrical power outages once a week.2
The average incidence of power outages in US hospitals is unknown as there is no centralized reporting system for hospital power failure events so true incidence is unknown.3
Causes (Developed Countries)3
Severe weather.
Hospital construction unmasks faults in internal electrical system
Power loss is frequently the result of failure of emergency generators and back up systems.

## Slide 4
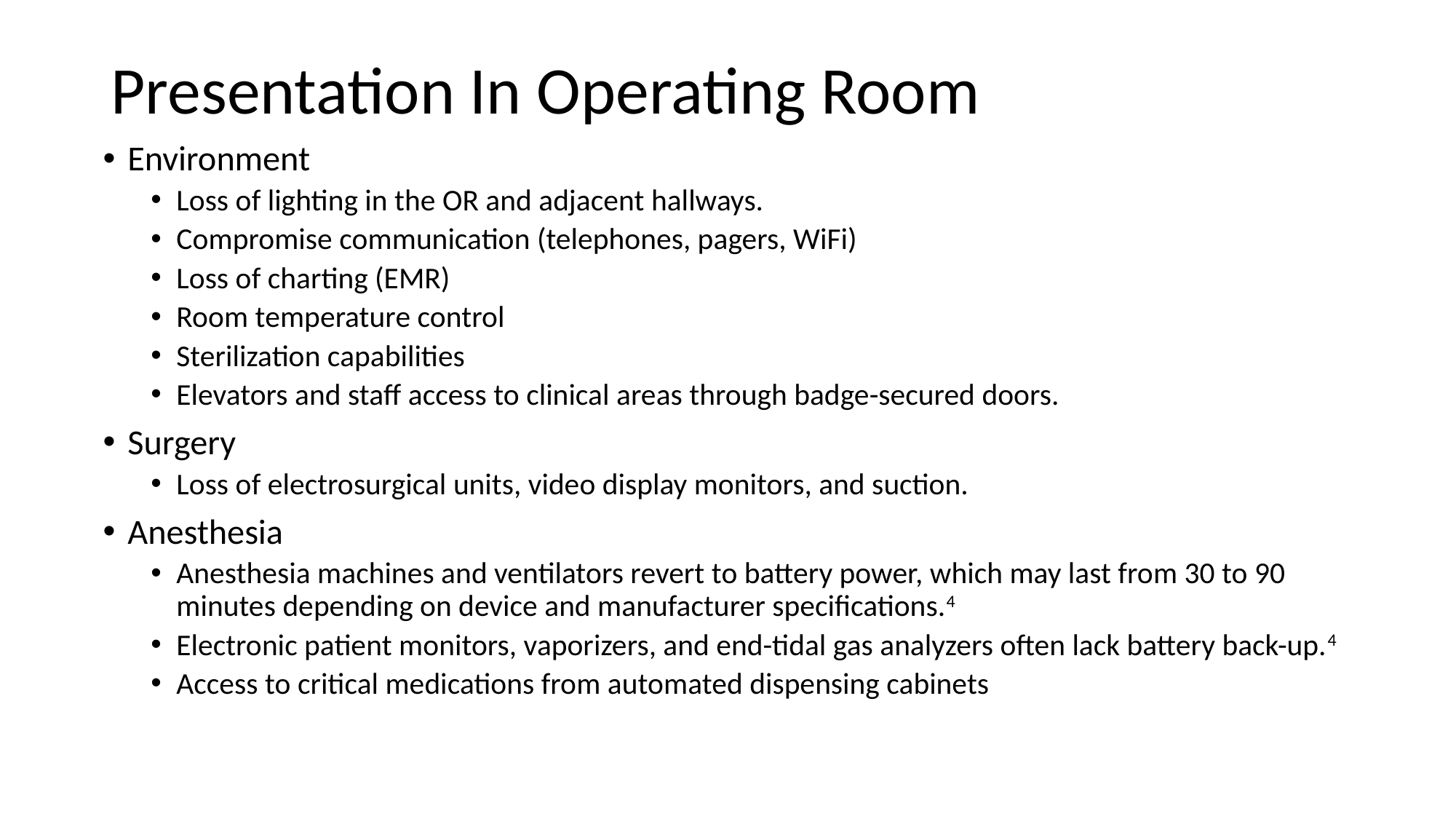

# Presentation In Operating Room
Environment
Loss of lighting in the OR and adjacent hallways.
Compromise communication (telephones, pagers, WiFi)
Loss of charting (EMR)
Room temperature control
Sterilization capabilities
Elevators and staff access to clinical areas through badge-secured doors.
Surgery
Loss of electrosurgical units, video display monitors, and suction.
Anesthesia
Anesthesia machines and ventilators revert to battery power, which may last from 30 to 90 minutes depending on device and manufacturer specifications.4
Electronic patient monitors, vaporizers, and end-tidal gas analyzers often lack battery back-up.4
Access to critical medications from automated dispensing cabinets

## Slide 5
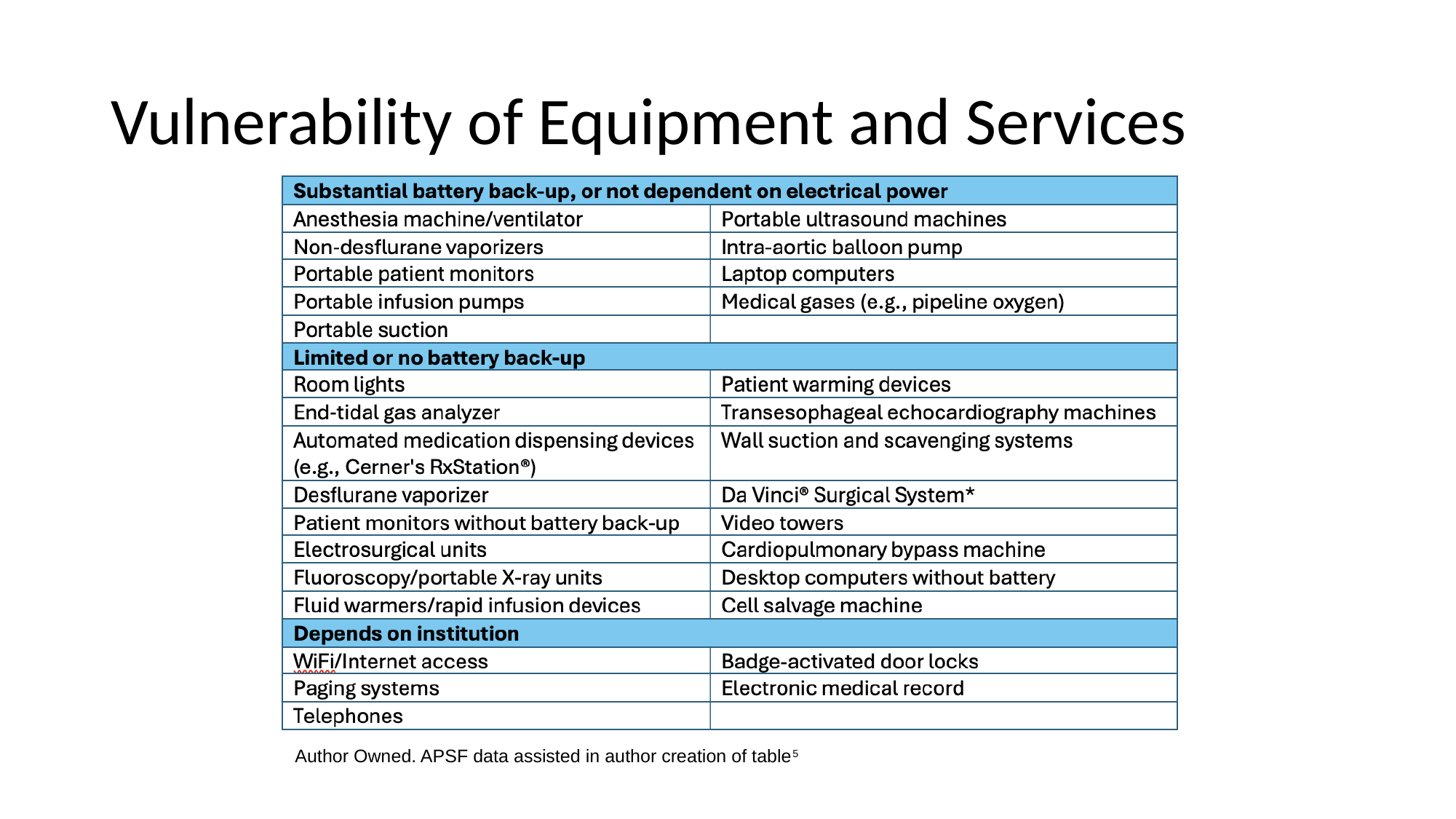

# Vulnerability of Equipment and Services
Author Owned. APSF data assisted in author creation of table5

## Slide 6
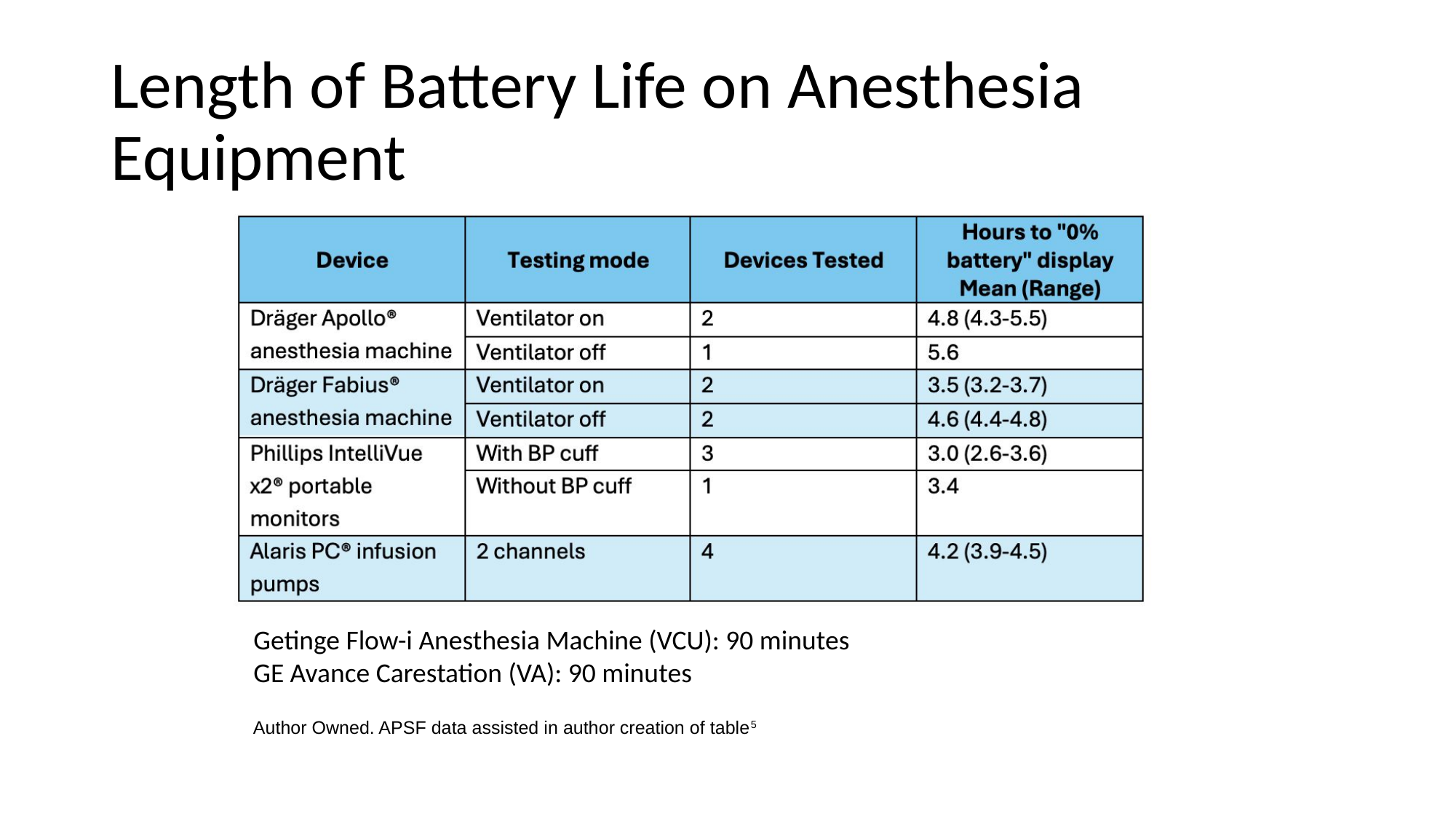

# Length of Battery Life on Anesthesia Equipment
Getinge Flow-i Anesthesia Machine (VCU): 90 minutes
GE Avance Carestation (VA): 90 minutes
Author Owned. APSF data assisted in author creation of table5

## Slide 7
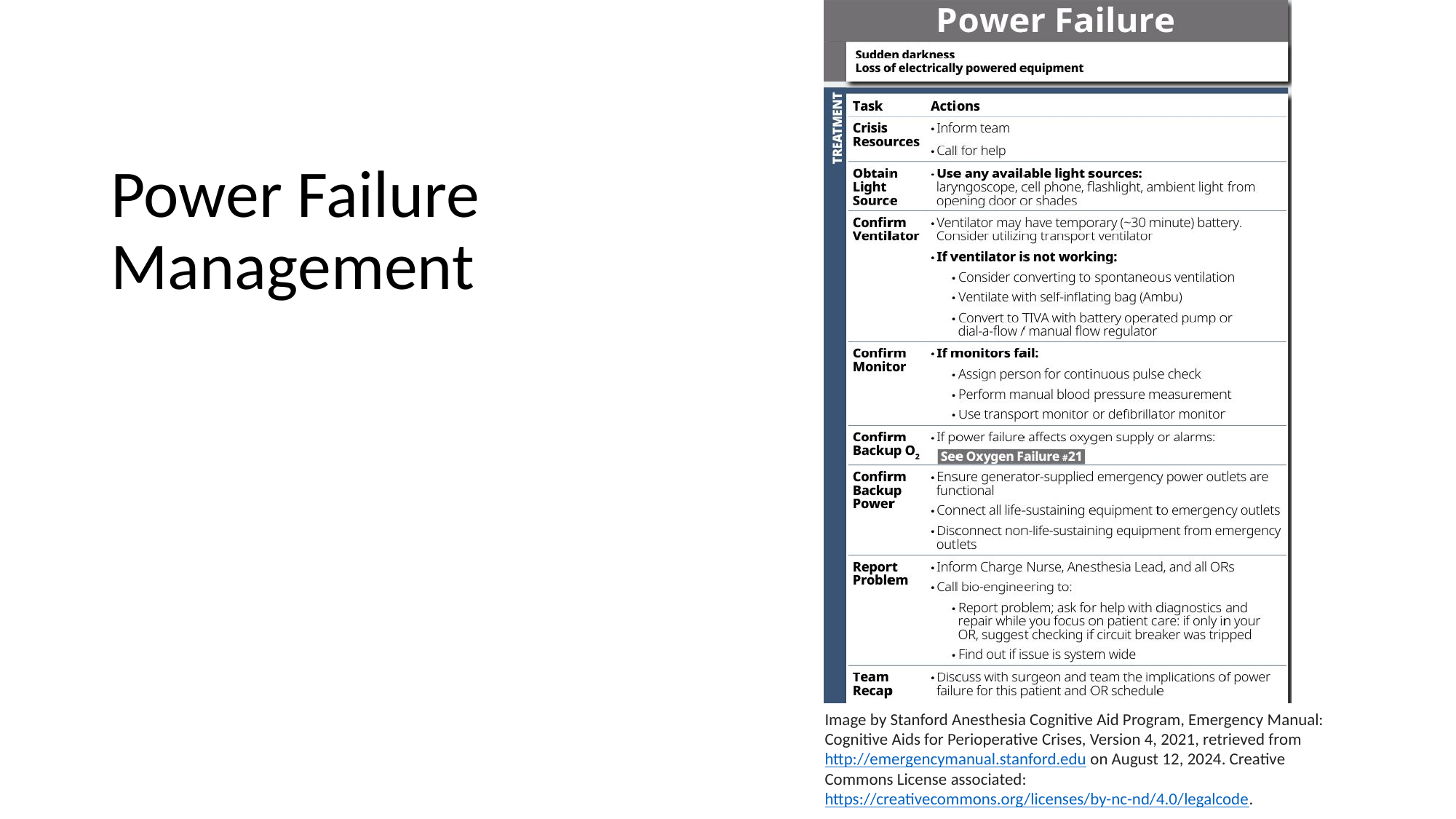

# Power Failure Management
Image by Stanford Anesthesia Cognitive Aid Program, Emergency Manual: Cognitive Aids for Perioperative Crises, Version 4, 2021, retrieved from http://emergencymanual.stanford.edu on August 12, 2024. Creative Commons License associated: https://creativecommons.org/licenses/by-nc-nd/4.0/legalcode.

## Slide 8
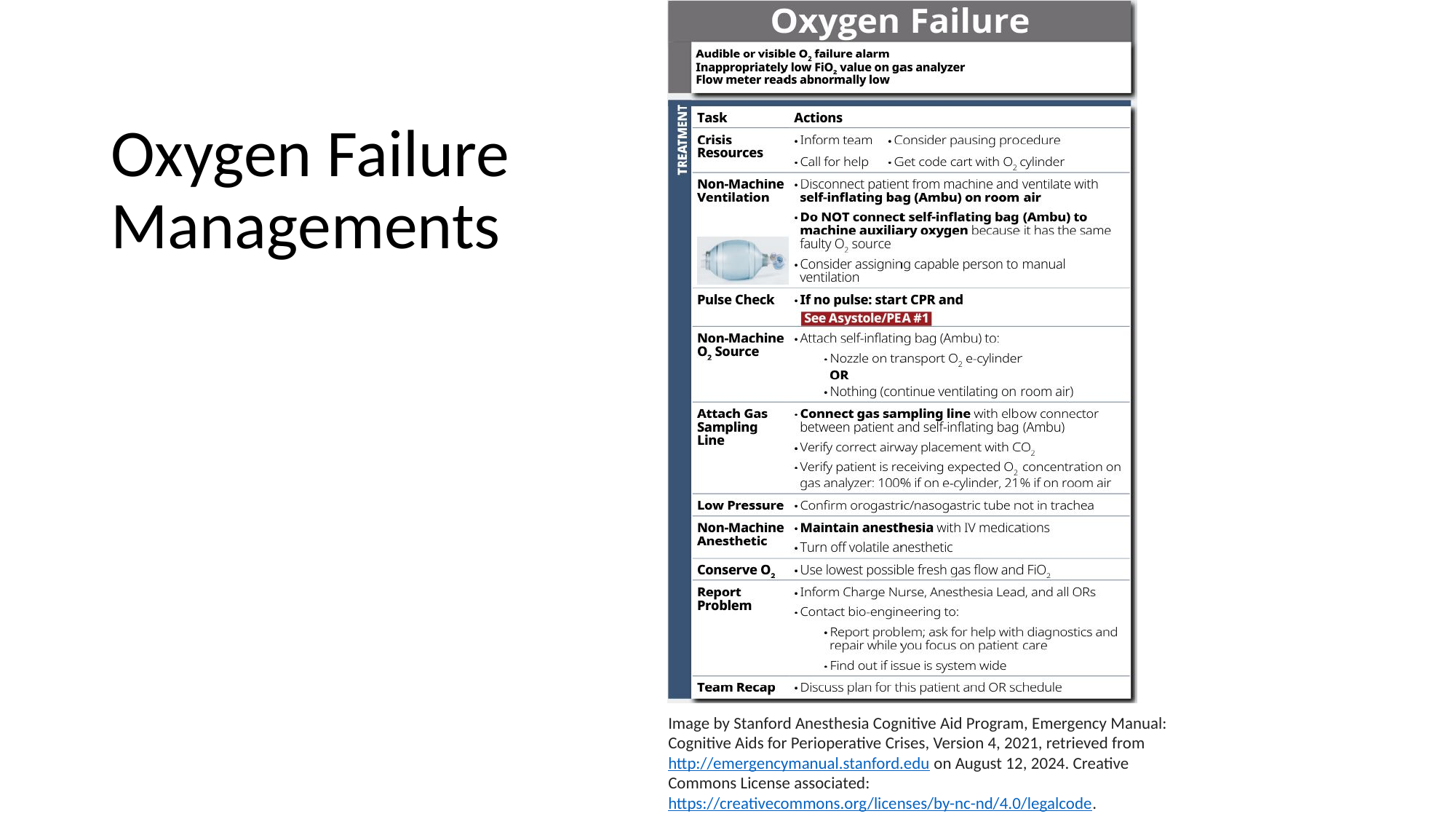

# Oxygen Failure Managements
Image by Stanford Anesthesia Cognitive Aid Program, Emergency Manual: Cognitive Aids for Perioperative Crises, Version 4, 2021, retrieved from http://emergencymanual.stanford.edu on August 12, 2024. Creative Commons License associated: https://creativecommons.org/licenses/by-nc-nd/4.0/legalcode.

## Slide 9
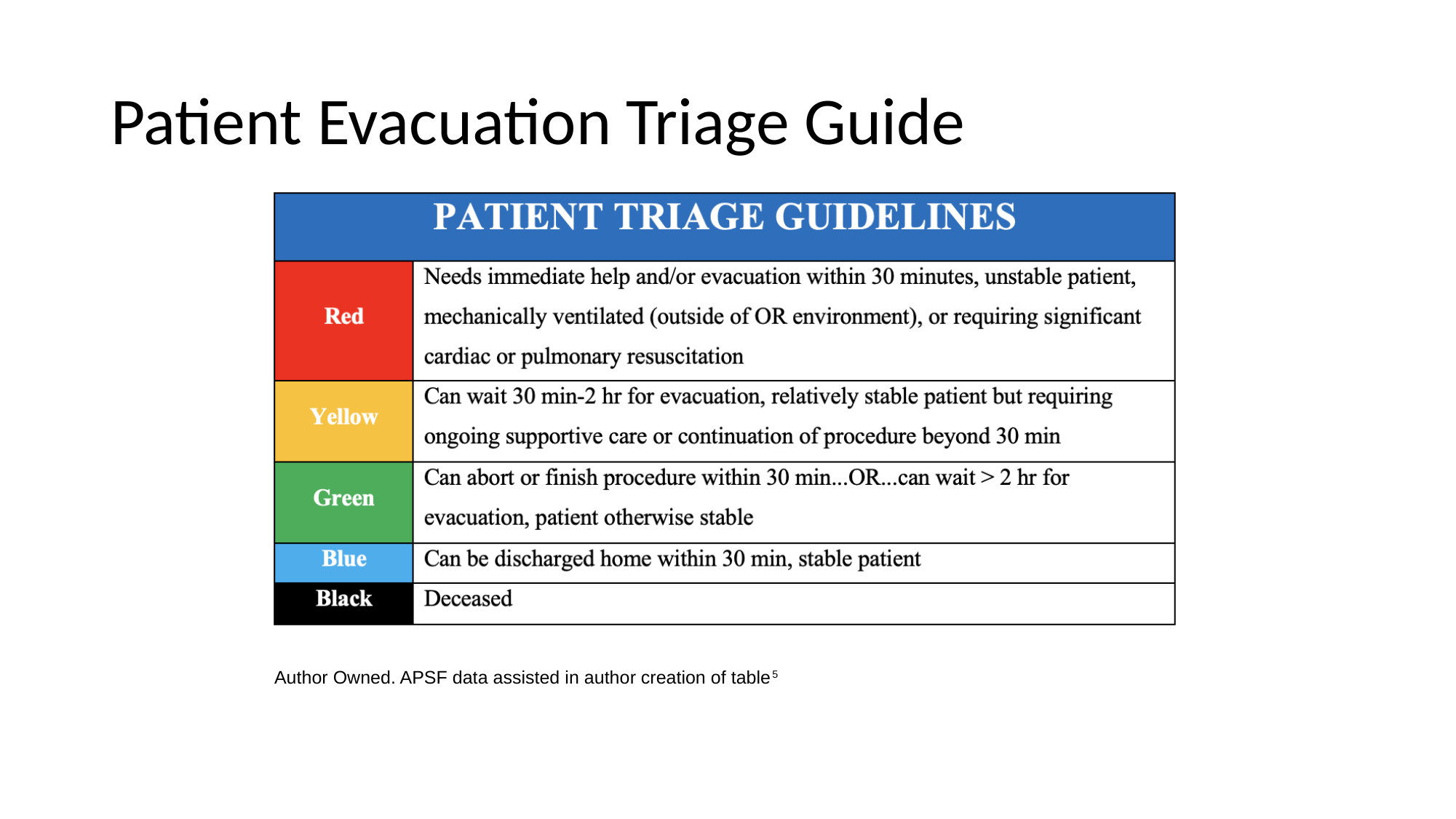

# Patient Evacuation Triage Guide
Author Owned. APSF data assisted in author creation of table5

## Slide 10
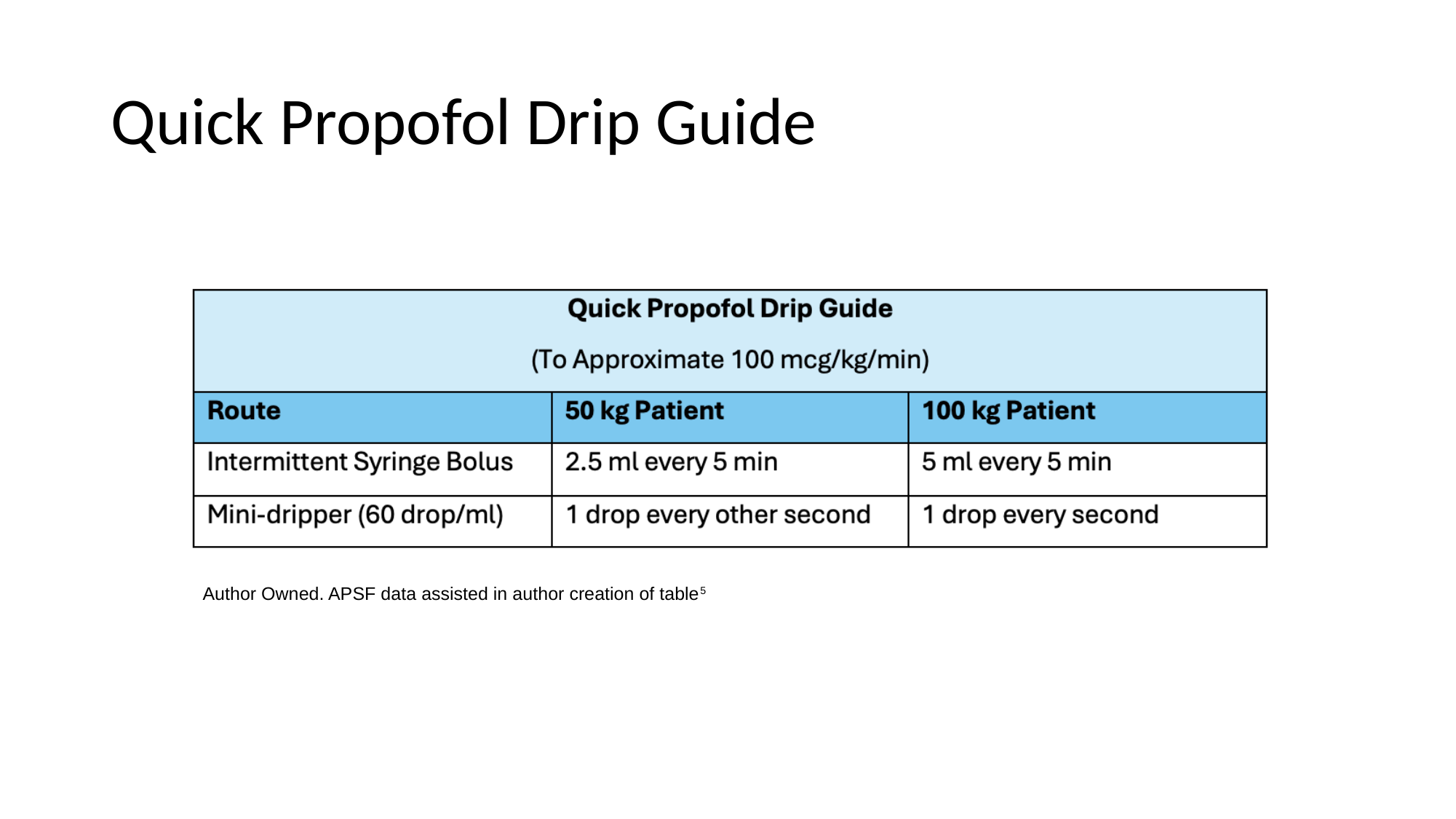

# Quick Propofol Drip Guide
Author Owned. APSF data assisted in author creation of table5

## Slide 11
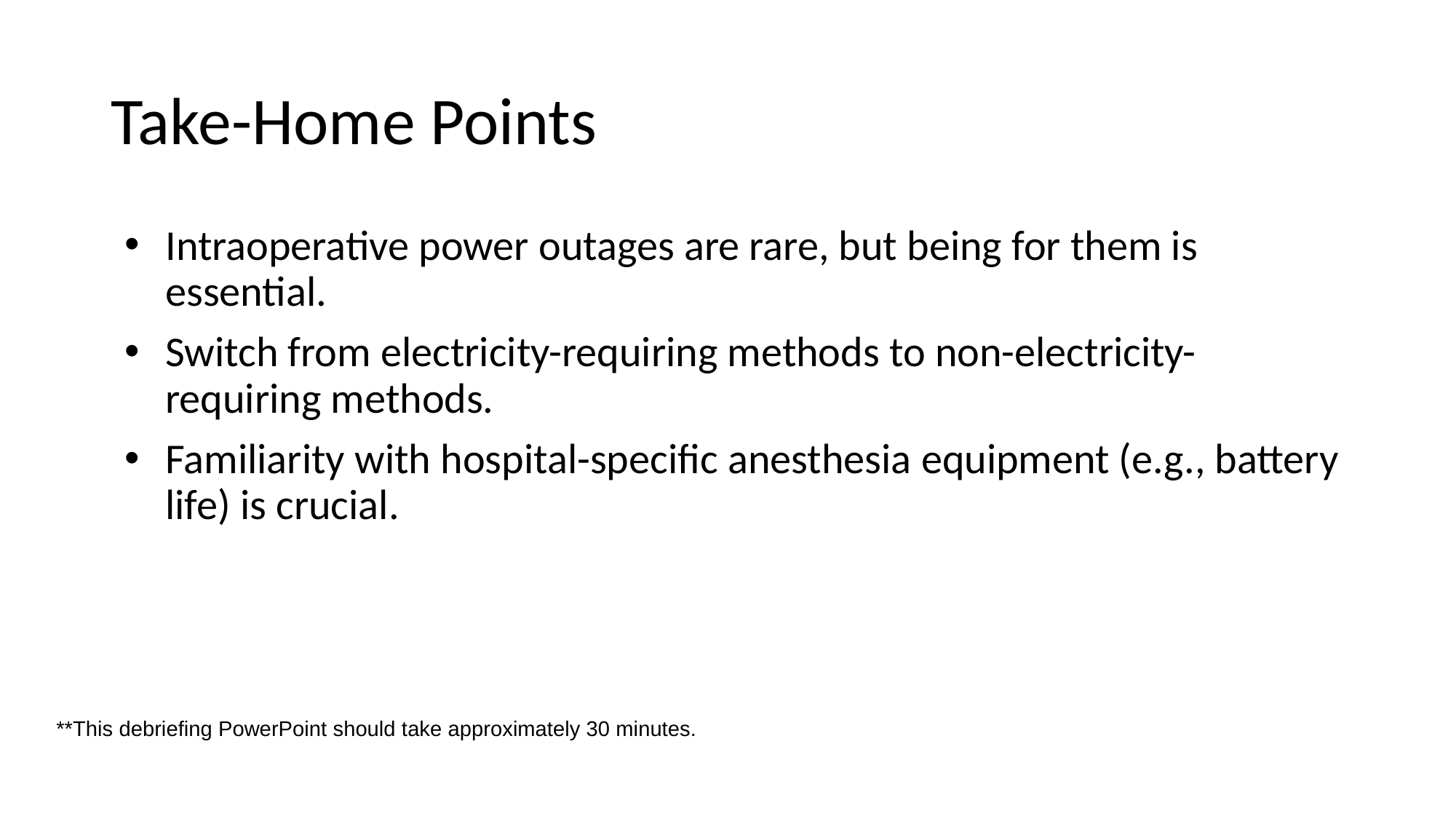

# Take-Home Points
Intraoperative power outages are rare, but being for them is essential.
Switch from electricity-requiring methods to non-electricity-requiring methods.
Familiarity with hospital-specific anesthesia equipment (e.g., battery life) is crucial.
**This debriefing PowerPoint should take approximately 30 minutes.

## Slide 12
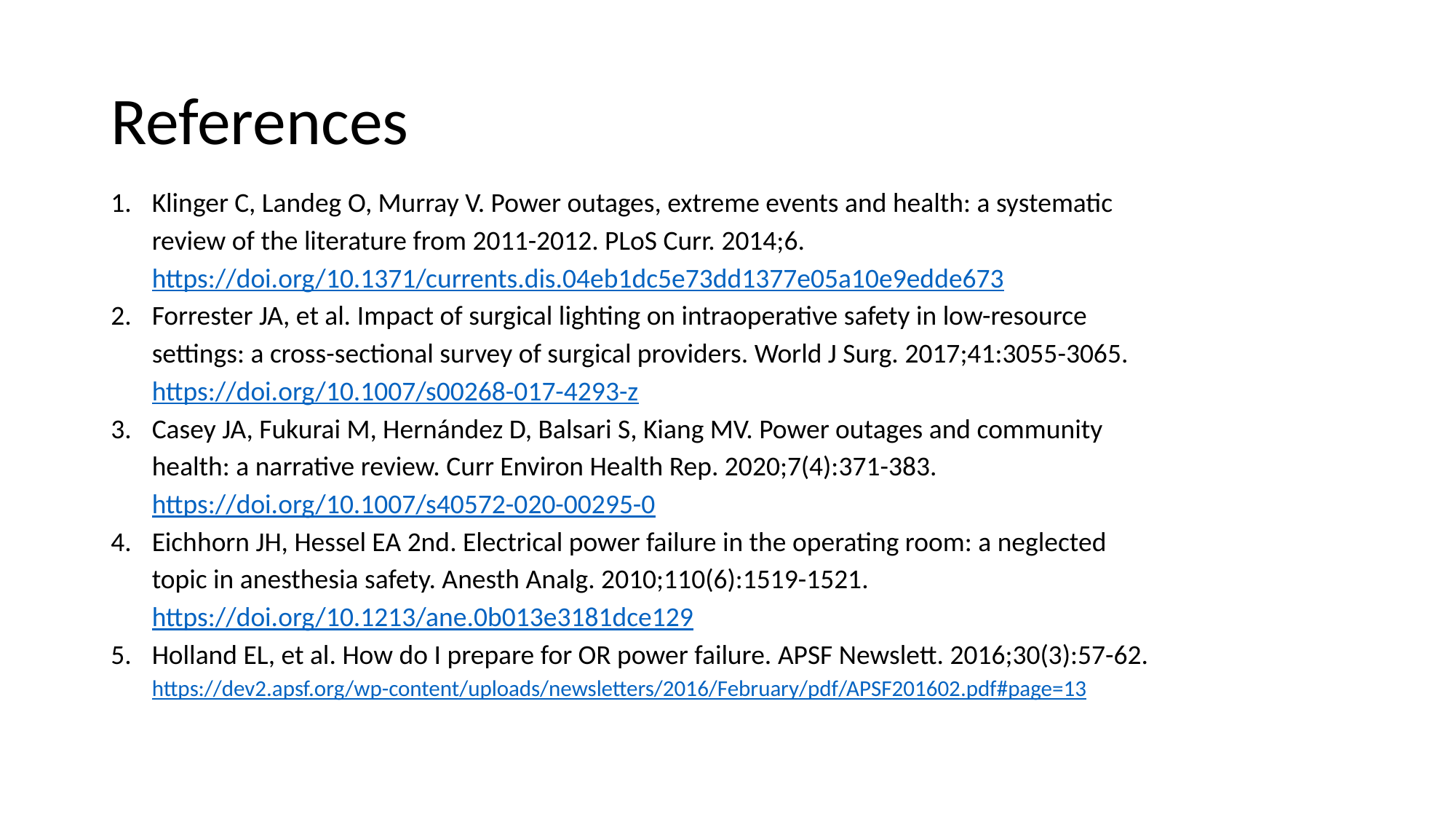

# References
Klinger C, Landeg O, Murray V. Power outages, extreme events and health: a systematic review of the literature from 2011-2012. PLoS Curr. 2014;6. https://doi.org/10.1371/currents.dis.04eb1dc5e73dd1377e05a10e9edde673
Forrester JA, et al. Impact of surgical lighting on intraoperative safety in low-resource settings: a cross-sectional survey of surgical providers. World J Surg. 2017;41:3055-3065. https://doi.org/10.1007/s00268-017-4293-z
Casey JA, Fukurai M, Hernández D, Balsari S, Kiang MV. Power outages and community health: a narrative review. Curr Environ Health Rep. 2020;7(4):371-383. https://doi.org/10.1007/s40572-020-00295-0
Eichhorn JH, Hessel EA 2nd. Electrical power failure in the operating room: a neglected topic in anesthesia safety. Anesth Analg. 2010;110(6):1519-1521. https://doi.org/10.1213/ane.0b013e3181dce129
Holland EL, et al. How do I prepare for OR power failure. APSF Newslett. 2016;30(3):57-62. https://dev2.apsf.org/wp-content/uploads/newsletters/2016/February/pdf/APSF201602.pdf#page=13
