# Supplementary material for: Power Outage: A Simulation Case for Anesthesiology Residents
Source: MedEdPORTAL. 2025 May 6;21:11523. doi: 10.15766/mep_2374-8265.11523 (PMC12052912; doi:10.15766/mep_2374-8265.11523)
Supplement: Supplementary file 1 — Simulation Case.docxSimulation Case Equipment.docxDebriefing Materials.pptxPostsimulation Survey.docx [file mep_2374-8265.11523-s001.zip › D. Postsimulation Survey.docx]

**Power Outage Simulation Evaluation Form**

| 1. I am a: 2. PGY-2 3. PGY-3 4. PGY-4 | Strongly  Disagree | Disagree | Neutral | Agree | Strongly  Agree |
| --- | --- | --- | --- | --- | --- |
| 1. This case presented during the simulation was appropriate for my level of education and training. | 1 | 2 | 3 | 4 | **5** |
| 1. The power outage simulation was a valuable learning experience. | 1 | 2 | 3 | 4 | **5** |
| 1. The simulation case was realistic. | 1 | 2 | 3 | 4 | **5** |
| 1. The power outage simulation was useful in developing my clinical reasoning and decision-making skills. | 1 | 2 | 3 | 4 | **5** |
| 1. The debrief promoted reflection and team discussion. | 1 | 2 | 3 | 4 | **5** |
| 1. The facilitators created an environment for discussion and exploration. | 1 | 2 | 3 | 4 | **5** |

Please rate your confidence regarding the following questions:

|  | Strongly Disagree | Disagree | Neutral | Agree | Strongly Agree |
| --- | --- | --- | --- | --- | --- |
| Before the simulation session, I could confidently manage a patient in the OR when there is a power outage. | 1 | 2 | 3 | 4 | **5** |
| After the simulation session, I could confidently manage a patient in the OR when there is a power outage. | 1 | 2 | 3 | 4 | **5** |
| Before the simulation session, I could confidently establish monitoring for vital signs for a patient when there is no power. | 1 | 2 | 3 | 4 | **5** |
| After the simulation session, I could confidently establish monitoring for vital signs for a patient when there is no power. | 1 | 2 | 3 | 4 | **5** |
| Before the simulation session, I feel I could confidently plan for disposition for patients when there is a power outage. | 1 | 2 | 3 | 4 | **5** |
| After the simulation session, I feel I could confidently plan for disposition for patients when there is a power outage. | 1 | 2 | 3 | 4 | **5** |

On a scale of 1-10 (1 being the worst, 10 the best), my faculty debriefer, Dr.________________, was effective. Comments:

How could we improve this simulation and workshop? Additional comments:
