# Supplementary material for: Power Outage: A Simulation Case for Anesthesiology Residents
Source: MedEdPORTAL. 2025 May 6;21:11523. doi: 10.15766/mep_2374-8265.11523 (PMC12052912; doi:10.15766/mep_2374-8265.11523)
Supplement: Supplementary file 1 — Simulation Case.docxSimulation Case Equipment.docxDebriefing Materials.pptxPostsimulation Survey.docx [file mep_2374-8265.11523-s001.zip › A. Simulation Case.docx]

| Appendix A: Simulation Case  SIMULATION CASE TITLE: Power Outage: A Simulation Case for Anesthesiology Residents  AUTHORS: Luke Johnson, BA, Ezoza Rajabaliev, DO, Kristin Canipe, RN, MSN, Michael Kazior, MD  LEARNER AUDIENCE: Anesthesiology residents | |
| --- | --- |
| PATIENT NAME: Johnny OSCE  PATIENT AGE: 62  CHIEF COMPLAINT: Right inguinal hernia  PHYSICAL SETTING: Operating room | |
|  | |
| Brief Narrative Description of Case | The patient is a 62-year-old male who presents for an elective open inguinal hernia repair. The patient has a history of hypertension, GERD, and depression. The learner will be taking over the case from a colleague under general endotracheal anesthesia. After treating a bronchospasm there will be a power outage. The participant will need to re-establish an anesthetic, monitor vital signs, maintain ventilation/oxygenation, and plan appropriate disposition. |
| Primary Learning Objectives | By the end of the simulation, the participant will be able to:  1.) Employ alternate light sources to establish visualization for surgery during a power outage.  2.) Use alternate methods to provide ventilation and oxygenation for the patient during a power outage.  3.) Switch from inhaled anesthetics to total intravenous anesthesia (TIVA) during a power outage.  4.) Use available alternative methods of monitoring the patient’s vital signs during a power outage.  5.) Effectively collaborate with other operating room personnel and physicians to create an appropriate disposition plan for the patient during a power outage. |
| Critical Actions | 1. Establish oxygenation by calling for an oxygen tank 2. Establish ventilation by switching from anesthesia machine to self-inflating bag valve mask. 3. Find appropriate method of monitoring vital signs (code cart, transport monitor, auscultation, etc). 4. Convert anesthetic from volatile anesthesia to total intravenous anesthesia via infusion pumps. 5. Find and use an alternative light source (laryngoscopes, cell phones, etc.) 6. Consult biomedical engineering (a team of professionals responsible for maintaining, repairing, and ensuring the safe operation of all medical equipment within a hospital), and alert anesthesia board runner. 7. Establish an appropriate disposition plan with the surgeon actor. |
| Pre-brief | 1. Welcome the learner and perform a pre-brief going over the following:    1. Outline of simulation events for the day.    2. Confidentiality rule.    3. Basic assumptions.    4. Commitment to creating a safe learning environment.    5. Fiction contract.    6. Debriefing.    7. Introduction to the simulation environment and scene safety.    8. Learner given case stem prior to beginning |

| Initial Presentation | | | |
| --- | --- | --- | --- |
| Initial Vital Signs | At the beginning of the simulation (patient already under general anesthesia):  Heart Rate: 70 BPM  Rhythm: Sinus  Blood Pressure: 130/90 mmHg  SpO2: 100%  Temperature: 36.5 C  Lung Sounds: Normal  EtCO2: 38  RR: 16 | | |
| Overall Setting and Appearance | Learners enter the operating room and take over for a colleague. The simulated operating room is equipped with real-time vital sign monitors for blood pressure, EKG, end-tidal carbon dioxide, respiratory rate, oxygen saturation, temperature, and arterial blood pressure. A glidescope, mock anesthesia cart with anesthesia disposables (ETT, laryngoscope blades, syringes, etc.) and an array of mock anesthesia medications including propofol and norepinephrine infusions. An adult code cart with a defibrillator were also present in the OR. The learner will encounter a Laerdal SimMan3G manikin representing the patient in this case who will appear stable. | | |
| Standardized Participants (and Their Roles in the Room at Case Start) | Role 1: Surgeon actress—no verbal script at beginning, continues with hernia repair which is initially going well | | |
| HPI | Patient is a 62-year-old male with hypertension presenting for a right inguinal hernia repair under general endotracheal anesthesia. The hernia was recently diagnosed. Patient also has a history of GERD and depression. | | |
| Past Medical/Surgical History | Medications | Allergies | Family History |
| Appendectomy  Tonsillectomy with adenoidectomy | Omeprazole  Lisinopril  Sertraline | No known drug allergies | None pertinent |
| Physical Examination | | | |
| General | Somewhat anxious, well-nourished and well-developed | | |
| Airway | MP 2, full neck range of motion, full dentition, mouth opening >3 finger breadths, TMD >6 cm | | |
| Lungs | Clear to auscultation bilaterally | | |
| Cardiovascular | No murmur, Regular rate and rhythm | | |
| Neurological | Alert and oriented. No focal deficits. | | |
| Induction of anesthesia | Medications: 180mg propofol, 100mg lidocaine, 100mcg fentanyl, 50mg rocuronium.  Airway: Easy mask, easy intubation. 7.0 oral ETT in place. | | |
| Intraoperative course | 1 PIV (20g) in right arm. Currently has 1 twitch. | | |

| Instructor Notes - Changes and CASE Branch Points | | |
| --- | --- | --- |
| Intervention / Time Point | Change in Case | Additional Information |
| 1. Baseline | Case begins with patient under general anesthesia.  Vital signs established | Heart rate: 70 bpm  Rhythm: NSR  Blood pressure: 130/90 mmHg  Respiratory rate: 16  SpO2: 100%  End-tidal CO2: 38 mmHg  Temperature: 36.5°C |
| 1. Mild hypotension | Hypotension will begin on instructor cue once the learner establishes a baseline understanding of the environment.  VS trend over 1 minute | Change to these vital signs:  HR: 94  BP: 88/55  RR: 16  SpO2: 98%  Temp: 36.5  CO2: 34 |
| 1. Resolution of hypotension | Learner will have to treat hypotension with fluid bolus and vasopressor administration to activate.  VS trend over 1 minute | Change to these vital signs:  HR: 100  BP: 112/66  RR: 16  SpO2: 98%  Temp: 36.5  CO2: 36 |
| 1. Advancing the Timeline by 30 Minutes | Starts on instructor cue once the learner has recognized stable vital signs. | Instructor will announce overhead:  “30 minutes have passed in the procedure. The patient has been stable in the interim and only required intermittent boluses of pressors to maintain the goal blood pressure. The operative case is going smoothly and the surgeon has announced that they will be closing in 20 minutes.” |
| 1. Hypoxia | Starts on instructor cue.  Patient starts to wheeze.  Participant should auscultate, identify a bronchospasm and start treatment.  Treatment can include increasing sevoflurane and/or administering albuterol.  VS trend over 1 minute. | Change to these vital signs:  HR: 100  BP: 108/62  RR: 16  SpO2: 92%  Temp: 36.5  CO2: 40 |
| 1. Power outage | Starts on instructor cue.  Vitals remain unchanged.  Sim operations specialist or facilitator cuts power to OR.  Participant will need to:   - Call for help. - Call for transport monitor or external defibrillator to monitor vitals. - Call for oxygen tank - Take off ventilator and move to BVM. - Switch anesthetic over to TIVA on infusion pumps. - Use other sources of light (phones, laryngoscopes, etc.) - Consult biomedical engineering. - Alert anesthesia board runner.   The hypoxia should resolve as they initiate the maneuvers to maintain safety in a power outage. This is at instructor preference. | Continue vital signs:  HR: 100  BP: 108/62  RR: 16  SpO2: 92%  Temp: 36.5  CO2: 40 |
| 7. Disposition | Begins on instructor cue.  Once all the items above have been completed, or at instructor cue, a conversation about disposition needs to occur between learners and surgeon actor.  If learners have not broached topic, the surgeon can cue with “Can we close and finish the case?”  Surgeon and learners should come together on a disposition plan.  Case ends. | Continue these vital signs:  HR: 100  BP: 108/62  RR: 16  SpO2: 98%  Temp: 36.5  CO2: 40 |

Ideal Scenario Flow

*The learners enter the simulated operating room and encounter a stable patient under general anesthesia for an elective inguinal hernia repair. After getting settled in the simulation, the learner encounters standard intraoperative hypotension which they should be able to treat quickly and effectively. The facilitator indicates 30 minutes have passed to get the operation close to the end of the procedure.*

*The learner will have to recognize a standard bronchospasm evidenced by hypoxia and wheezing on auscultation. This serves to distract the learner while the standardized patient or other personnel cut power to the simulated OR. We used a switch that cut all power to the room other than the manikin so the learners could still auscultate the lungs, palpate pulses, control the simulator, etc. Our anesthesia machine went to backup power so we had to have the surgeon actor manually turn the machine off when the power went out. The hypoxia will improve during this time period so the learners focus on treating the power outage, not the bronchospasm*

*The learner will have to meet all the requirements above to safely monitor the patient and maintain general anesthesia.*

- *When the learner calls for an oxygen tank, it can be brought to the room by the facilitator, sim tech, or other personnel.*
- *When the learner calls for a bag valve mask, it should be hanging on the back of the anesthesia machine for them to find.*
- *To convert to a total IV anesthetic, the infusion pumps should already be in the room.*
- *When they call for a different monitor there are several options. We couldn’t use the travel monitor connected to the anesthesia machine because it can’t read the manikin vitals. We brought in an extra monitor that displayed the manikin vitals when they asked for a travel monitor. Another option is the manual external defibrillator on the code cart, which will register the ECG from the manikin with the right connection.*
- *When they consult biomedical engineering and call the board runner, they have a phone they use that connects to the control room. The facilitator or other personnel answers and asks as that particular consult.*

*Once all the above tasks are complete, they will need to work with the surgeon to establish an appropriate disposition. The surgeon is close to completing the surgery so the case is set up to get the learners to convince the surgeon to finish and then extubate in the operating room. The rationale is that this is an elective case on a relatively healthy patient who should require relatively little resources if extubated at the end of the case and allowed to go home, rather than leaving them intubated and requiring mechanical ventilation in the post-anesthesia care unit (PACU) which may also not have power. These resources need to be saved for other patients that will need them more (more complex cases or sicker patients).*

*Once the plan is agreed upon, the case will end.*

Anticipated Management Mistakes

1. Hypotension & hypoxia: If given to senior anesthesia residents, it is doubtful they will have difficulty recognizing and managing these routine issues. More junior residents may have issues with recognizing the bronchospasm.
2. Mobilizing vital signs monitoring: Of all the different management goals, this is the hardest to accomplish in the simulation center. It can be difficult to assess what they can use in the simulated OR and what they cannot. Especially since the anesthesia machine has to be manually turned off. Some of our learners had difficulty identifying the best way to monitor vitals and simply decided to put a hand on the pulse and look for hypoxia with skin changes. They may need to be prompted to ask for a separate travel monitor or defibrillator, at which point the separate monitor with vitals can be brought in
3. Disposition Plan: Learners commonly try to keep the patient intubated with their first disposition plan. The surgeon is prompted to try to convince them to extubate them, however, if the learners persist with their initial plan the surgeon is told to not protest too heavily. The learner must then organize the PACU vent and will sometimes realize that they should extubate.
4. Difficulty managing multiple tasks simultaneously: During the power outage, learners will need to manage ventilation, monitor vital signs, communicate with team members, and plan for patient transfer all at once. This can be overwhelming, and learners may struggle to prioritize and manage these tasks efficiently.
