# Supplementary material for: Power Outage: A Simulation Case for Anesthesiology Residents
Source: MedEdPORTAL. 2025 May 6;21:11523. doi: 10.15766/mep_2374-8265.11523 (PMC12052912; doi:10.15766/mep_2374-8265.11523)
Supplement: Supplementary file 1 — Simulation Case.docxSimulation Case Equipment.docxDebriefing Materials.pptxPostsimulation Survey.docx [file mep_2374-8265.11523-s001.zip › _Educational Summary Report_11523.pdf]

MedEdPORTAL® | The AAMC Journal of Teaching and Learning Resources

Original Publication Open Access

Power Outage: A Simulation Case for Anesthesiology Residents

Luke Johnson*, Ezoza Rajabaliev, DO, Kristin Canipe, RN, MSN, Michael R. Kazior, MD

*Corresponding author: johnsonlm12@vcu.edu

Abstract

Introduction: Power outages in the OR are rare. However, anesthesia providers must be prepared to manage these situations until power is restored or their patient can be moved to a safe area. These situations occur so infrequently that many learners do not experience these events during their training. We designed a high-fidelity power outage simulation for anesthesiology residents to fill this training gap and enhance their preparedness and confidence. **Methods:** In each simulation session consisting of up to four learners, one or two PGY 3/PGY 4 residents participated as anesthesiologists in a case involving an intraoperative power loss during a routine inguinal hernia repair of a patient under general anesthesia. After the simulation, residents received a debriefing focused on intraoperative power outage training. After concluding the debriefing, residents completed a 5-point Likert scale survey to assess their confidence in managing an intraoperative power loss. **Results:** Over 2 years, 22 anesthesiology residents completed the simulation. Residents' mean ratings of confidence in managing a patient in the OR during a power outage improved by 1.2 points ($p =.001$), confidence in monitoring vital signs improved by 1.4 ($p = .001$), and confidence in planning appropriate disposition improved by 0.9 ($p =.001$). All participants found the simulation highly valuable. **Discussion:** The anesthesiology-specific simulation proved to be an effective educational tool. Feedback was positive as residents agreed that the simulation was valuable for developing clinical reasoning and decision-making skills, significantly boosting their confidence to respond effectively and maintain patient safety.

Keywords
Simulation, Power Outage, Anesthesiology, Critical Care Medicine

Educational Objectives

By the end of the activity, learners will be able to:

1. Employ alternate light sources to establish visualization for surgery during a power outage.
2. Use alternate methods to provide ventilation and oxygenation for the patient during a power outage.
3. Switch from inhaled anesthetics to total intravenous anesthesia (TIVA) during a power outage.
4. Use available alternative methods of monitoring the patient's vital signs during a power outage.
5. Effectively collaborate with other OR personnel and physicians to create an appropriate disposition plan for the patient during a power outage.

Citation:
Johnson L, Rajabaliev E, Canipe K, Kazior MR. Power outage: a simulation case for anesthesiology residents. *MedEdPORTAL*. 2025;21:11523. https://doi.org/10.15766/mep_2374-8265.11523

Introduction

In the US, intraoperative power outages are rare but there are notable examples of occurrences.[1] In the OR, power outages can disrupt crucial life-supporting systems and pose serious threats to patients under general anesthesia. In the event of a power outage, the anesthesiologist must ensure that critical life-supporting systems continue to function or immediately initiate appropriate backup measures. Thus, it is essential for anesthesiologists to be trained in these emergency responses. However, evidence suggests that anesthesia providers receive inadequate training to prepare them to manage anesthesia delivery during a power outage.[2]

Simulation scenarios are a common and effective way that anesthesiologists are trained during residency.[3,4] Perioperative crises such as airway fire, malignant hyperthermia, and local anesthetic systemic toxicity have many resources and simulations dedicated to managing them; however, very few educational resources exist for managing intraoperative power outages, as this aspect of training is often overlooked.[5,6] The uniqueness of power outage situations necessitates more focused training, which is evidenced by a report indicating that

Copyright © 2025 Johnson et al. This is an open-access publication distributed under the terms of the Creative Commons Attribution license.

most anesthesia providers are interested in receiving further training.[2]

Addressing this training gap, we created a high-fidelity simulation case of an intraoperative power outage. We designed the simulation using Ericksson's deliberate practice and Schon's reflective practice conceptual frameworks.[7,8] Deliberate practice guided the structure, while reflective practice encouraged residents to analyze their simulation experience. We tailored the simulation for upper-level anesthesiology residents, since they may be better equipped to handle complex scenarios. Currently, no simulation cases similar to this have been reported in *MedEdPORTAL* or other published literature, highlighting the importance of this simulation.

During an intraoperative power outage, there are specific steps for anesthesiologists to prevent, manage, and mitigate the effects.[9] Our simulation prepares residents to take these steps to keep patients safe during power outages. Many residency programs have implemented anesthesia crisis simulation curricula,[10] and we suggest adding this scenario to further incorporate conceptual frameworks of medical education such as deliberate practice and reflective practice.[11]

Simulating a scenario in which the backup battery of the ventilator fails is both important and practical. Though most modern ventilators in the US have backup power, learners may participate in medical missions during their careers in settings that do not have ventilators with backup power. Further, even in the US, backup power will eventually expire, resulting in the situation portrayed in the simulation. Regarding practicality, if there was backup power, there would be essentially no decisions to make during the simulation.

Methods

Development

We developed this scenario (Appendix A) as one of many cases for anesthesiology residents in a perioperative crises high-fidelity simulation–based curriculum at the Virginia Commonwealth University (VCU) School of Medicine Center for Human Simulation & Patient Safety. This power outage simulation provided training specifically for upper-level (PGY 3 and PGY 4) resident training. The initial development process included an anesthesiology attending, a research resident, and the simulation center educator. Together, these individuals created the case stem, identified the necessary supplies and equipment for the simulation, and wrote the debriefing questions. We designed the case stem to be realistic, to challenge the experienced PGY 3 and PGY 4 residents to think critically and apply their knowledge. No specific learner prerequisites exist.

Equipment/Environment

This scenario occurred in a simulated OR at an academic simulation center. We set the scene with the high-fidelity manikin on the OR table with one large bore IV access connected to fluids and already placed on monitors. The anesthesia machine had already been checked, and the room was stocked with all necessary equipment and supplies. Appendix B contains a list of the equipment we found necessary to run this simulation successfully.

Personnel

One anesthesiology faculty member was present to act as an instructor for the simulation and debriefing. A simulation operation specialist was present to manage the patient simulator and technology. A standardized patient given a script acted as the surgeon during the simulation, though we recommend an attending or resident surgeon for this role. During our stimulation sessions, we were unable to secure an attending surgeon for this role due to clinical demands. Given our circumstances, scheduling a standardized patient was more feasible to ensure consistent availability throughout the sessions. While an attending surgeon would have been ideal, we equipped the standardized patient with set speaking lines to prepare them for the scenario, and then communicated with them via an earpiece to deliver answers to questions in real time. A simulation educator was also present to provide technical assistance for learners and staff during the session and set up the simulation scenario appropriately.

Implementation

One to four learners participated in each simulation session, consisting of a total of 17 PGY 3 anesthesiology residents and five PGY 4 anesthesiology residents. One to two residents per session were chosen to be the learners serving as anesthesiologists in the simulation.

The scenario (Appendix A) begins with a prebriefing lasting between 5 and 10 minutes, which includes a presentation of the case stem by a faculty facilitator. During the prebriefing, learners discover that the case involves managing the general anesthesia for a 62-year-old male patient undergoing a right inguinal hernia repair. The case had already begun and learners would be taking over for a colleague partway through the case.

After the prebriefing, the one or two learners serving as anesthesiologists enter the OR, while the other learners watch

Copyright © 2025 Johnson et al. This is an open-access publication distributed under the terms of the Creative Commons Attribution license.

from the debriefing room via video. The primary learners have the ability to call for help and at any point may have the other learners watching come in and join them.

To ensure that the participants are comfortable and engaged in the simulation scenario, the learners are first instructed to treat the patient for routine, postinduction hypotension. After learners appropriately address the hypotension, the facilitator indicates that 30 minutes have passed, in which the intraoperative course was uneventful.

The patient then develops hypoxia secondary to bronchospasm, providing a distraction in the scenario. As the learners begin management, the simulation technician activates a switch that turns off the overhead lights and power to the anesthesia machine, but power to the 3G manikin remains. This simulates a power outage while still being able to manipulate the manikin. The loss of power to the anesthesia machine does not override the emergency backup, and therefore the simulation surgeon positions themselves near the anesthesia machine to manually turn it off. The simulation lasts 20–25 minutes.

Key actions to take include switching to bag-valve mask, calling for a full oxygen tank, calling for a portable monitor for vital signs, and converting to total intravenous anesthesia. Required consults include biomedical engineering and the anesthesia board runner to plan for the potential transfer of the patient to a location with power. Patient disposition must be agreed upon between the participant and the surgeon. The surgeon is told to guide the participants toward extubating and going to the postanesthesia care unit; however, it is ultimately left up to the participant. Participants had to accomplish all of these tasks while utilizing alternative light sources, such as laryngoscopes or cell phones. After these main interventions are achieved and a destination for the patient is established, the scenario ends.

After the simulation, learners participated in a 30-minute debriefing session using a debriefing guide (Appendix C). Learners then completed the postsimulation survey (Appendix D).

Debriefing

Once the simulation was completed, all learners, those participating and those watching, went to a separate room with the instructor for debriefing. Learners had the chance to reflect upon their actions during the simulation case and recognize areas to be improved upon. In addition, learners were aided in developing strategies to better address a power outage crisis. The debriefing materials (Appendix C), which include the Stanford Medicine Emergency Manual Guide, are meant to guide this conversation and elicit discussion with learners.

Assessment

With the educational objectives in mind, we designed a 5-point Likert scale survey (1= *strongly disagree;* 5 = *strongly agree;* Appendix D).

The first survey section contained questions about simulation appropriateness for training level and overall quality of the simulation. Next, the survey asked residents to self-assess their ability to manage a patient, confidently monitor vital signs, and plan for patient disposition in the absence of power in the OR, according to our educational objectives. We designed the survey to provide insight into the learners' strengths and weaknesses in these areas.

Moreover, the assessment asked participants to rate their self-reported comfort level and competence in managing a power outage, both before and after the simulation.

The survey concluded by asking open-ended questions about how the simulation could be improved and any additional comments. Residents completed the surveys after the educational debriefing session.

After collecting data, we conducted a two-tailed paired *t*test to compare the pre- and postsimulation confidence scores (mean and SD) evaluating the participating residents' confidence level in meeting the intended educational objectives.

Institutional review board approval was not obtained. However, all residents received the same education; therefore, no extra risk existed in this educational curriculum. To ensure anonymity, residents were instructed not to include their names or any identifiable information on the surveys, maintaining data confidentiality throughout both the presimulation and postsimulation analysis.

Results

This simulation scenario was run nine times on 3 separate days over a 2-year period with a total of 22 participating residents. The participants were five PGY 4 anesthesia residents (23%) and seventeen PGY 3 anesthesia residents (77%).

As indicated in the Figure, the simulation greatly augmented residents' confidence in all three measured aspects. In comparing pre- and postimulation confidence levels, the residents' confidence in managing a patient with a power outage improved by 1.2 points ($p = .001$), confidence in their ability to monitor a

Copyright © 2025 Johnson et al. This is an open-access publication distributed under the terms of the Creative Commons Attribution license.

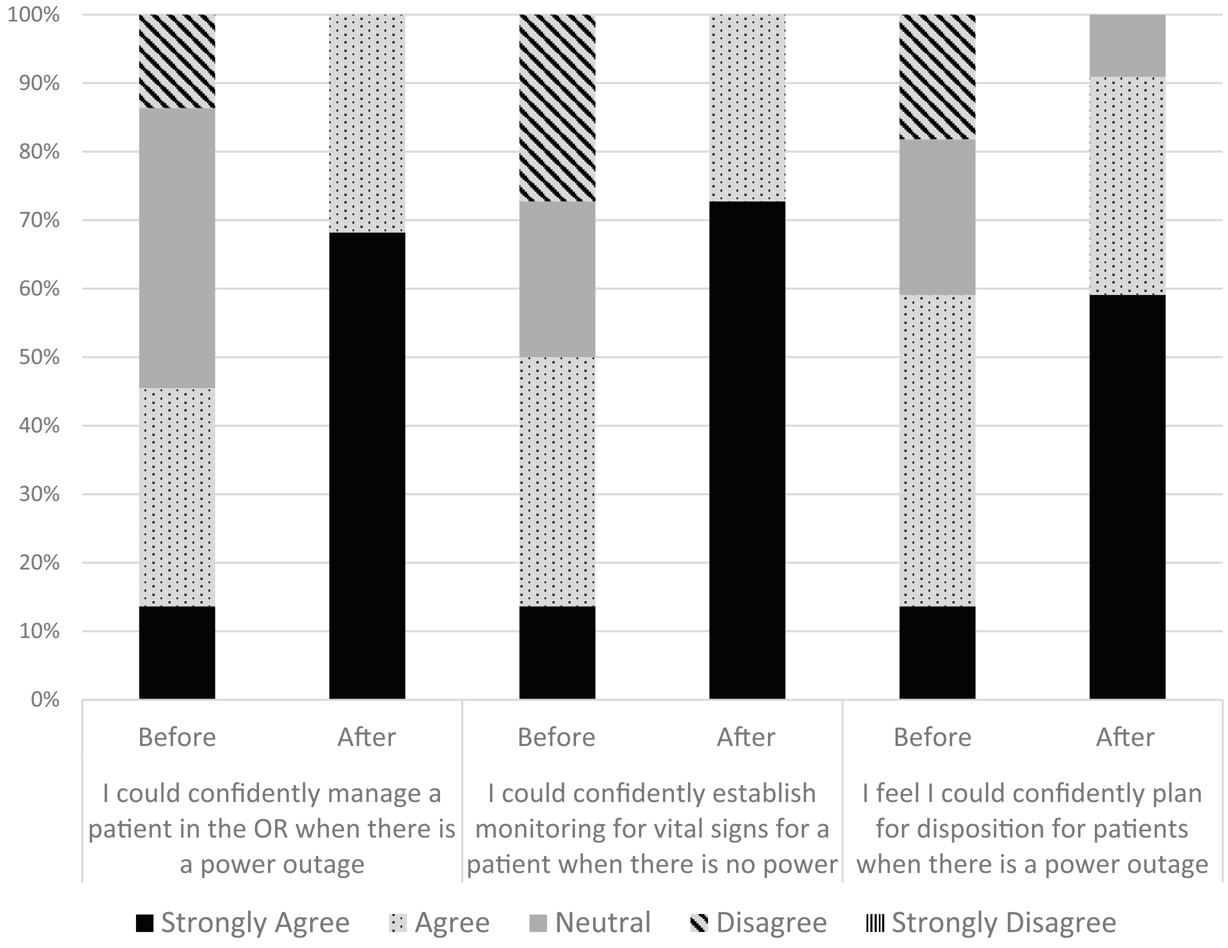

Figure. Distribution of anesthesiology residents' responses to questions related to confidence levels on the power outage simulation survey, rated on a 5-point Likert scale before and after the simulation ($N = 22$). Abbreviation: OR, operating room.

patient's vital signs improved by 1.4 ($p = .001$), and confidence in planning appropriate disposition improved by 0.9 ($p = .001$).

When asked if the simulation was appropriate to their training level, all 22 learners agreed or strongly agreed that it was. All learners also strongly agreed that the simulation was a valuable learning experience. In the open-ended question portion of the survey, one resident described the simulation as "very helpful" in their clinical training. Two residents indicated that they would have preferred a shorter simulation with the power outage occurring sooner.

Discussion

This novel simulation for upper-level anesthesiology residents improved participants' confidence in managing a patient, monitoring vital signs, and planning appropriate disposition during an intraoperative power outage.

This simulation scenario helps add more hands-on practice exercise for anesthesia providers to help prepare for a power outage. Potential participants for this simulation would be attending anesthesiologists, certified registered nurse anesthetists, and other anesthesia providers. ORs could use this as a basis for team training with other specialties to prepare for an event like this. Although there are many case reports of intraoperative power outages in which best practices are outlined, this educational experience represents the first evidence of the benefit of offering a power outage simulation to anesthesia learners. Our findings contribute and align with the well-documented benefit of simulation in anesthesia education.

After initial implementation of this scenario, learner feedback indicated that some elements were less relevant to the primary educational objectives related to power outages. One area that

Copyright © 2025 Johnson et al. This is an open-access publication distributed under the terms of the Creative Commons Attribution license.

the upper-level residents said they thought was superfluous was the induction of anesthesia and intubation at the beginning of the case. Their opinion was that it made the scenario unnecessarily longer and depleted time to focus on managing the power outage. A longer simulation time also makes the debriefing time shorter. After the first year, this part was removed from the simulation scenario.

A technical aspect that still needs to be optimized is the ventilator management during the simulation. The anesthesia machines at our institution have a battery backup that turned on when the power was cut to the simulated OR. The simulation surgeon had to manually turn the machine off when the lights went out, to prevent the learners from continuing to use the device. We also had to remind the learners in the simulation session that they could not turn the machine back on. Future directions include working with the manufacturer to see if the anesthesia machine in the simulation center can have its backup battery modified.

While this project provides insight into the educational innovation being evaluated, readers should consider certain limitations. First, the small sample size of participants from one institution may limit the generalizability of the findings. Second, the evaluation relied solely on self-reported measures, which may not accurately reflect participants' actual performance.

Considering these limitations, future research could explore the impact of this educational intervention on the performance of anesthesia residents within the clinical environment. This could include collecting additional outcome types, such as directly observed performance measures, standardized tests, or assessment of clinical practice, to provide a more comprehensive understanding of the simulation's effects, or conducting objective performance assessments, such as standardized tests or direct observation of clinical practice, to gain a more accurate understanding of the effects of the simulation. Such research could help identify areas for improvement and refine the educational intervention to better meet the needs of anesthesia residents.

Future education based on this power outage scenario may include a focused review of the specifics of the anesthesia machine. Many of the questions that arose in the debriefing were related to the different fail-safe mechanisms built into the machine. This is certainly an area where there is a large need for further education for anesthesia personnel as the devices have become much more technologically advanced over the last decade.

Appendices

A. Simulation Case.docx

B. Simulation Case Equipment.docx

C. Debriefing Materials.pptx

D. Postsimulation Survey.docx

All appendices are peer reviewed as integral parts of the Original Publication.

Luke Johnson: Third-Year Medical Student, Virginia Commonwealth University School of Medicine;
ORCID: https://orcid.org/0009-0000-1243-9852

Ezoza Rajabaliev, DO: Third-Year Resident, Department of Anesthesiology, Virginia Commonwealth University School of Medicine

Kristin Canipe, RN, MSN: Simulation Educator, Center for Human Simulation and Patient Safety, Virginia Commonwealth University School of Medicine

Michael R. Kazior, MD: Assistant Professor, Department of Anesthesiology, Virginia Commonwealth University Health; Staff Physician, Department of Anesthesiology, Richmond VA Medical Center

Acknowledgments
We thank Johnafaye Mariano, who assisted with writing the manuscript.

Disclosures
None to report.

Funding/Support
None to report.

Ethical Approval
Reported as not applicable.

References

1. Yasny J, Soffer R. A case of a power failure in the operating room. *Anesth Prog*. 2005;52(2):65-69. https://doi.org/10.2344/0003-3006(2005)52[65:ACOAPF]2.0.CO;2
2. Hayanga HK, Barnett DJ, Shallow NR, et al. Anesthesiologists and disaster medicine: a needs assessment for education and training and reported willingness to respond. *Anesth Analg*. 2017;124(5):1662-1669. https://doi.org/10.1213/ANE.0000000000002002
3. Chang CH. Medical simulation is needed in anesthesia training to achieve patient's safety. *Korean J Anesthesiol*. 2013;64(3):204-211. https://doi.org/10.4097/kjae.2013.64.3.204
4. Lighthall GK. The value of simulation training during anesthesia residency. *Anesthesiology*. 2006;105(2):433. https://doi.org/10.1097/00000542-200608000-00037

Copyright © 2025 Johnson et al. This is an open-access publication distributed under the terms of the Creative Commons Attribution license.

5. Eichhorn JH, Hessel EA II. Electrical power failure in the operating room: a neglected topic in anesthesia safety. *Anesth Analg*. 2010;110(6):1519-1521. https://doi.org/10.1213/ANE.0b013e3181dce129

6. Smith CR, Peng YG. The evolution and role of simulation in medical education. *APSF Newsl*. 2021;36(2):82-84.

7. Ericsson KA. Deliberate practice and the acquisition and maintenance of expert performance in medicine and related domains. *Acad Med*. 2004;79(10)(suppl):S70-S81. https://doi.org/10.1097/00001888-200410001-00022

8. Schön DA. *The Reflective Practitioner: How Professionals Think in Action*. Routledge; 2017. https://doi.org/10.4324/9781315237473

9. Ortega J, Quesada J, Yi C. Disaster preparedness: a checklist for massive power outage during surgery. Zenodo. January 1, 2022. Accessed April 2, 2025. https://doi.org/10.5281/zenodo.6653483

10. Wilcocks K, Kapralos B, Uribe Quevedo A, Alam F, Dubrowski A. The anesthesia crisis scenario builder for authoring anesthesia crisis-based simulations. *IEEE Trans Games*. 2020;12(4):361-366. https://doi.org/10.1109/TG.2020.3003315

11. Zackoff MW, Real FJ, Abramson EL, Li STT, Klein MD, Gusic ME. Enhancing educational scholarship through conceptual frameworks: a challenge and roadmap for medical educators. *Acad Pediatr*. 2019;19(2):135-141. https://doi.org/10.1016/j.acap.2018.08.003

Received: August 12, 2024
Accepted: March 31, 2025
Published: May 6, 2025

Copyright © 2025 Johnson et al. This is an open-access publication distributed under the terms of the Creative Commons Attribution license.
